# Thrombus Trifecta: A Laparoscopic Sleeve Gastrectomy Complication

**DOI:** 10.7759/cureus.29506

**Published:** 2022-09-23

**Authors:** Clates P Adams, Andrew Zabel, Vanessa Hannick

**Affiliations:** 1 Transitional Year Residency, William Beaumont Army Medical Center, Fort Bliss, USA; 2 Emergency Medicine, Carl R. Darnall Army Medical Center, Fort Hood, USA

**Keywords:** low molecular weight heparin, tips, portal vein thrombosis, abdominal pain, sleeve gastrectomy

## Abstract

We describe a case of generalized, extreme, colicky abdominal pain status post laparoscopic sleeve gastrectomy where the patient formed thromboses in the portal vein, superior mesenteric vein, and splenic vein, which were visualized with computed tomography (CT) imaging. The case was managed using the standard of care, which is anticoagulation and/or surgical intervention, both of which were used in this case.

## Introduction

Laparoscopic sleeve gastrectomy (LSG) involves removing the greater curvature and fundus of the stomach; the partial gastrectomy is oriented vertically, parallel to the lesser curvature of the stomach. The procedure causes weight loss in a restrictive manner [1]. The portal vein is formed by the confluence of the splenic and superior mesenteric veins, which drain the spleen and small intestine, respectively. Occlusion of the portal vein by a thrombus typically occurs in patients with acquired conditions (such as cirrhosis, hepatocellular carcinoma, myeloproliferative disorders, antiphospholipid syndrome, paroxysmal nocturnal hemoglobinuria, and recent pregnancy) and/or prothrombotic disorders (such as factor V Leiden, prothrombin gene mutations, protein C or S deficiency, and antithrombin deficiency).

## Case presentation

A 40-year-old male presented to the emergency department, with pain and vomiting, status post uncomplicated LSG two weeks prior. The patient endorsed severe mild epigastric abdominal pain, worsening over two days, inability to eat without immediately vomiting, and several bouts of non-bloody diarrhea. Vital signs were within normal limits and pain was rated to be 10/10. The patient appeared alert and oriented, visibly anxious, and in mild distress. His abdomen was diffusely tender, with severe epigastric tenderness and mild ascites.

**Figure 1:**
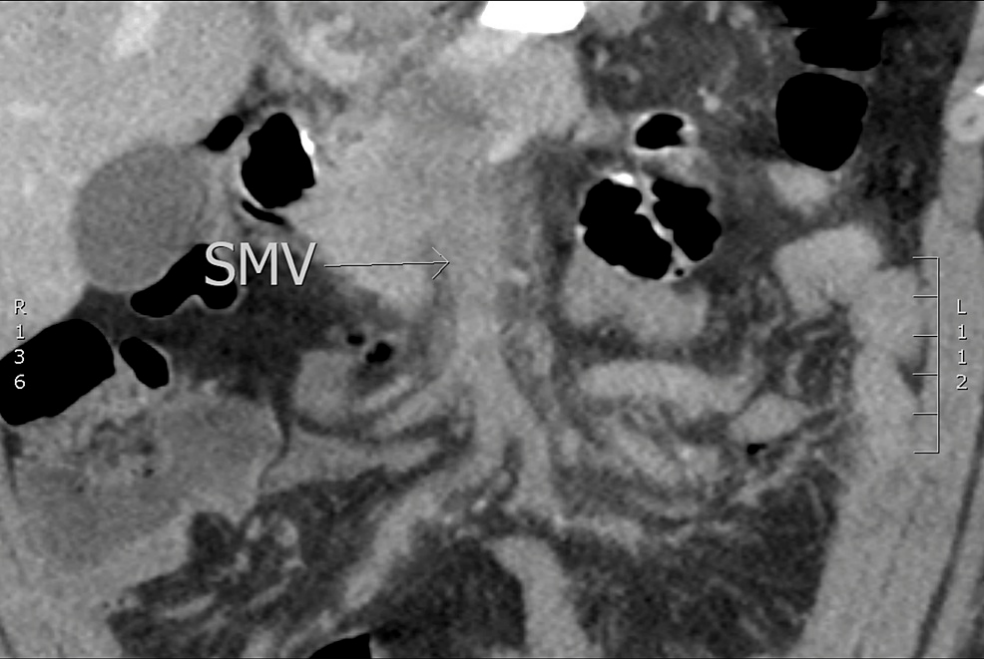
Thrombophlebitis of the superior mesenteric vein and draining mesenteric branches SMV, superior mesenteric vein.

**Figure 2:**
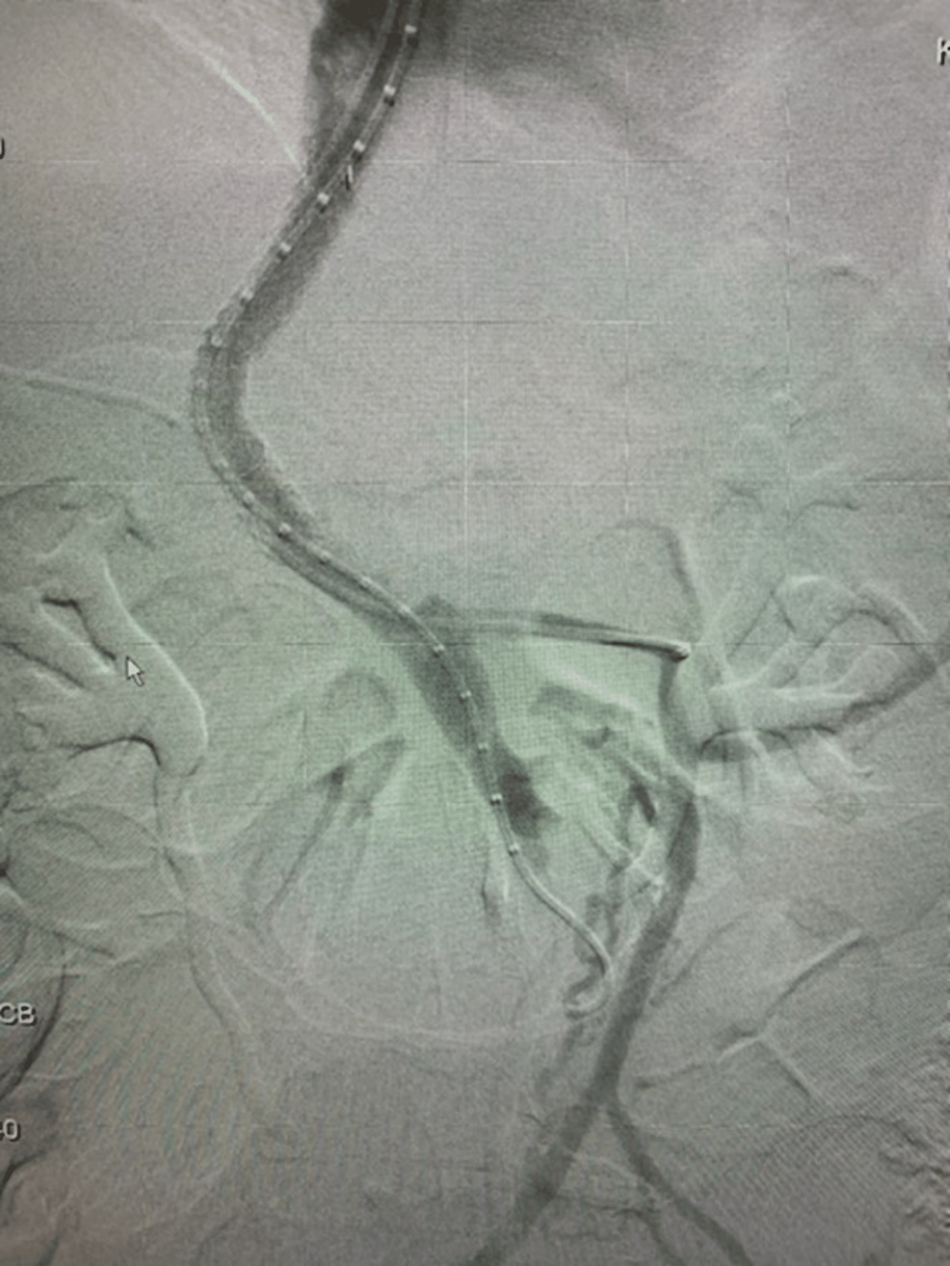
Transjugular intrahepatic portosysetmic shunt procedure performed with fluoroscopy

**Figure 3:**
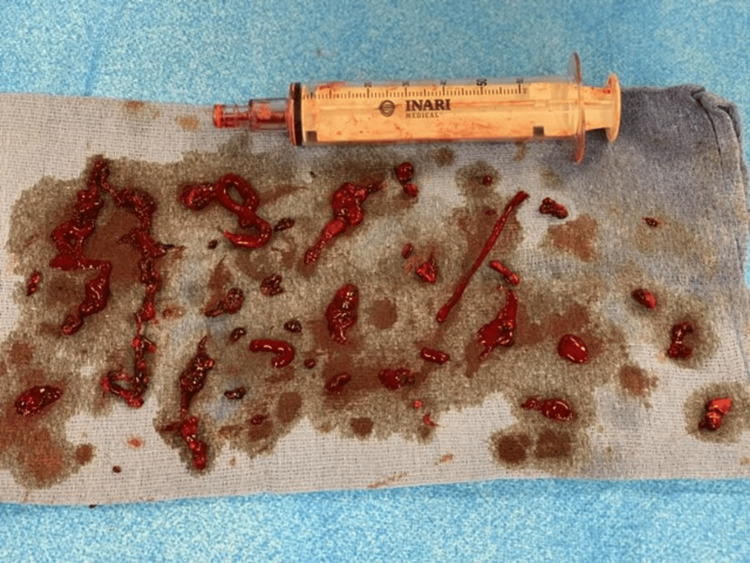
Removed clot burden

In this case, computed tomography (CT) abdomen and pelvis with IV and oral contrast (per local bariatric protocol due to recent LSG) revealed complete portal vein thrombosis (PVT), partial splenic vein thrombosis, and superior mesenteric vein thrombosis (Figure 1). The patient was anticoagulated with low-molecular-weight heparin (LMWH) and admitted to medicine. Two days later, he was transferred to a regional medical center surgical service, due to clot burden and worsening liver function tests, for vascular surgery intervention. A transjugular intrahepatic portosystemic shunt (TIPS) procedure was performed (Figure 2), and a large amount of the clot was removed (Figure 3). Follow-up testing for inherited clotting disorders was negative. 

## Discussion

Although it is rare after bariatric surgery, PVT is most common after LSG. In a study of over 5700 patients who participated in laparoscopic bariatric surgery, 17 had thrombosis, 16 of whom underwent a sleeve gastrectomy [2]. 

In a study of almost 24,000 autopsies in Sweden performed from 1970 to 1982, the prevalence of PVT was 1% without LSG [3]. PVT decreases hepatic venous outflow while preserving arterial inflow, which can lead to complications such as intestinal ischemia, portal hypertension, and portal cholangiopathy [4]. Liver tests, as with this patient, are typically normal because hepatic arterial blood flow compensates for decreased portal inflow [5].

The primary management of acute PVT is anticoagulation and, when possible, treatment of predisposing conditions. Anticoagulation is recommended with LMWH. Several case reports have documented successful lysis of acute PVT using streptokinase or tissue plasminogen. Other successful modalities include thrombectomy via TIPS procedure [6]. TIPS placement reduces elevated portal pressure by creating a low-resistance channel between the hepatic vein and an intrahepatic branch of the portal vein. The typical indication is portal hypertension with complications such as variceal bleeding and ascites. TIPS is typically performed by interventional radiology. 

## Conclusions

Abdominal pain is one of the most common complaints in the emergency department. The threshold for obtaining a CT, in these patients, is often low. However, in the case of recent abdominal surgery, the threshold to obtain a CT scan should be even lower. Although rare, PVT as well as the involvement of the splenic and superior mesenteric veins may occur in patients without the aforementioned risk factors. It is prudent to obtain an accurate medical history and perform a physical examination in all patients to be able to render the best medical care possible. 
